# Outcomes of hepatocellular carcinoma patients treated in the lenvatinib and immunotherapy era (2018–2021) compared to the sorafenib era (2008–2018)

**DOI:** 10.1002/cam4.7415

**Published:** 2024-07-02

**Authors:** Chloe A. Lim, Carla P. Amaro, Philip Q. Ding, Winson Y. Cheung, Vincent C. Tam

**Affiliations:** ^1^ Internal Medicine Residency Program, Cumming School of Medicine University of Calgary Calgary Alberta Canada; ^2^ Tom Baker Cancer Centre University of Calgary Calgary Alberta Canada

**Keywords:** hepatocellular carcinoma, lenvatinib and atezolizumab with bevacizumab, outcomes of HCC treatment, sorafenib and other treatments

## Abstract

**Background:**

Lenvatinib (LEN) and atezolizumab + bevacizumab (A + B) have drastically changed the treatment paradigm for advanced hepatocellular carcinoma (HCC). Before these landmark trials, sorafenib (SOR) served as the standard first‐line treatment for a decade. Our study aimed to assess the outcomes of HCC patients treated during the SOR era (2008–2018) in contrast to those in the post‐SOR era (2018–2021), of which the predominant first‐line treatments were LEN or A + B.

**Methods:**

Inclusion criteria of the study were all HCC patients in the Canadian province of Alberta who started first‐line systemic therapy at cancer centers between 1 January 2008 and 31 December 2021. Survival outcomes, including overall survival (OS) and progression‐free survival (PFS), along with clinician‐assessed response rate (RR), were subject to retrospective analysis.

**Results:**

Of 372 total patients, 230 received treatment in the SOR era and 142 in the post‐SOR era. The demographic and clinical characteristics for the SOR era and post‐SOR era groups are as follows, respectively: the median age was 63 and 64 years, 80% and 81% were male, and 24% and 11% were of East Asian ethnicity. Before receiving systemic treatment, 40% and 33% received TACE, 7% and 9% received TARE, and 3% and 14% received SBRT in the two eras, respectively. In the post‐SOR era, patients received A + B (23%), LEN (51%), and SOR (23%) as first‐line treatment. There was a statistically significant improvement in RR (15% vs. 26%; *p* = 0.02), median PFS (3.8 months vs. 7.9 months; *p* < 0.0001), and median OS (9.8 months vs. 17.0 months; *p* < 0.0001).

**Conclusions:**

In this retrospective multicenter real‐world study, HCC patients treated in the post‐SOR era, where LEN and A + B were commonly used first‐line treatments, exhibited superior OS, PFS, and RR compared to patients treated in the SOR era. The findings of this study affirm the tangible progress achieved in the real world in enhancing outcomes for HCC patients through advancements in treatments over the past 15 years.

## 1 INTRODUCTION

Hepatocellular carcinoma (HCC) stands as the most prevalent primary malignancy affecting the liver and ranks as the third leading cause of cancer‐related mortality globally.^1^ Despite well‐established guidelines for HCC screening in chronic liver disease population, incidence continues to rise globally.^2^ Although local therapies have a role in the treatment of HCC, the majority of patients are diagnosed at an advanced stage, necessitating systemic therapy as the primary treatment approach.

Sorafenib (SOR), an oral multityrosine kinase inhibitor, has stood as the standard first‐line treatment for advanced HCC not amenable to local therapy for approximately a decade, spanning from 2008 to 2018 based on the SHARP trial.^3^ The study showed that median survival was improved to 10.7 months with SOR compared to 7.9 months median survival with placebo. Another phase 3 trial of HCC patients from the Asia‐Pacific region also showed that SOR improves overall survival (OS) compared to placebo.^4^

Recently, a number of clinical trials have shown improved efficacy of newer treatments when compared to SOR. In 2018, lenvatinib (LEN) was approved by Health Canada based on noninferiority of OS compared to SOR, improved progression‐free survival (PFS), response rate (RR) and better tolerability.^5^ The recent IMbrave150 trial examined the first‐line combination of atezolizumab, a PD‐L1 inhibitor, and bevacizumab, an antivascular endothelial growth factor antibody. This phase 3 trial showed that compared to SOR, atezolizumab, and bevacizumab (A + B) significantly improved median OS (13.4 months vs. 19.2 months), median PFS (4.3 months vs. 6.9 months), and overall response rate (ORR; 11% vs. 30%).^4^ Other first‐line treatments showing a survival benefit over SOR in clinical trials, including STRIDE (durvalumab plus tremelimumab) and camrelizumab plus rivoceranib, were not publicly funded in Canada at the time of this study.^6^, ^7^ With these advances in first‐line therapy options, as well as the emergence of second‐line TKI treatment options including cabozantinib and regorafenib,^8^, ^9^ it is imperative to examine real‐world outcomes and determine if these treatments have translated into meaningful improvement in outcomes for HCC patients over time. Previous retrospective studies explored outcomes comparing different first‐line therapies^10^, ^11^ and in Canada we have published data on outcomes with SOR and LEN treatment.^12^, ^13^ To the best of our knowledge, there are no studies that have systematically compared real‐world outcomes of advanced HCC patients receiving various treatments over time.

This study retrospectively evaluated outcomes and characteristics of unresectable, advanced HCC patients treated with systemic therapy in the SOR era versus post‐SOR era in a multi‐centre real‐world population.

## 2 METHODS

### 2.1 Study population

All patients with a confirmed diagnosis of Barcelona Clinical Liver Cancer (BCLC) stage B or C HCC and who received first‐line systemic therapy between December 2008 and December 2021 at cancer centers in the Canadian province of Alberta were retrospectively identified from the cancer care pharmacy database.

### 2.2 Study data

The electronic medical record (EMR) was reviewed and patient demographics, clinical and pathological data, treatment data, and outcome characteristics were collected.

Patients were classified into two different groups based on the time of systemic treatment initiation. SOR has been the standard of care first‐line systemic therapy for advanced HCC since 2008 until August 2018 when LEN was first available to Canadian patients. In accordance with this criterion, patients who initiated a first‐line systemic treatment from January 1, 2008 to July 31, 2018, were categorized as being treated in the “SOR era.” Patients who started first‐line systemic treatment from 1 August 2018 to 31 December 2021 were categorized as being treated in the “post‐SOR era.”

For each patient, all prior treatment modalities were collected. These include surgery, stereotactic body radiation (SBRT), tumor ablation, or tumor embolization (trans‐arterial chemoembolization [TACE], trans‐arterial radioembolization [TARE]). Liver function prior to and after treatment was evaluated by the Child–Pugh class score and albumin‐bilirubin (ALBI) score. Best radiographic response was recorded as per oncologist's clinical assessment in medical note as well as imaging reports from local radiologists.

The study protocol was approved by the Health Research Ethics Board of Alberta (HREBA. CC‐15‐0042).

### 2.3 Statistical analyses

The baseline characteristics of the overall population, as well as those of the SOR era and post‐SOR era subgroups, were summarized using descriptive statistics. Continuous variables were expressed as median and interquartile range, while categorical variables were presented as frequency and proportion. To compare subgroups for continuous variables, the Wilcoxon rank‐sum test was employed, and for categorical variables, Pearson's chi‐square test or Fisher's exact test was utilized. OS and PFS were estimated using the Kaplan–Meier method, and differences were assessed using the log‐rank test.

All statistical tests were two‐sided and a *p* < 0.05 was considered statistically significant. Statistical analysis was performed using R Core Team (2023). R: A Language and Environment for Statistical Computing. R Foundation for Statistical Computing, Vienna, Austria. https://www.R‐project.org/.

## 3 RESULTS

### 3.1 Description of study population

A total of 372 patients with BCLC B or C HCC and who received first‐line systemic therapy were identified and included in this study. Of these 230 patients were classified as SOR era and 142 patients classified as post‐SOR era. Baseline patient characteristics are summarized in Table 1. Overall cohort median age at systemic treatment start was 64 years, with SOR era median age of 63 and post‐SOR era median age of 65 years. There was 11% of East Asian ethnicity in post‐SOR cohort in comparison to SOR cohort of 24%. For etiology of liver disease, a smaller proportion of hepatitis B was seen in the post‐SOR era (15% compared to 27% in SOR era). Distribution of better Eastern Cooperative Oncology Group Performance Status (ECOG PS) and Child–Pugh class, BCLC stage, and ALBI grade was noted in post‐SOR group. A larger proportion of patients received SBRT and TARE as well as tumor ablation in post‐SOR era; conversely, higher proportion of patients received TACE and liver transplant in SOR era.

**TABLE 1:** Demographic and clinical characteristics.

Demographic	SOR era (*n* = 230)	post‐SOR era (*n* = 142)	*p*‐value
Median age (years)	63	65	0.06
Sex			
Male	185 (80%)	115 (81%)	0.90
Female	45 (20%)	27 (19%)	
Ethnicity			
East Asian	54 (24%)	15 (11%)	**0.002**
Non‐East Asian	176 (76%)	126 (89%)	
Etiology of liver disease			
Excess alcohol use	55 (24%)	42 (30%)	0.23
Hepatitis C	85 (37%)	68 (48%)	**0.04**
Hepatitis B	62 (27%)	21 (15%)	**0.006**
NASH	27 (12%)	18 (13%)	0.79
Tumor thrombus/macrovascular invasion	90 (40%)	46 (32%)	0.17
Main portal vein invasion	39 (17%)	10 (7%)	**0.006**
Endoscopy within 6 months of first‐line treatment	38 (17%)	42 (30%)	**0.003**
Localized treatment prior to systemic treatment			
TACE	74 (32%)	28 (20%)	**0.009**
TARE	16 (7%)	13 (9%)	0.44
SBRT	6 (3%)	12 (9%)	**0.01**
Liver transplant	15 (7%)	6 (4%)	0.35
Tumor ablation	55 (24%)	37 (26%)	0.30
ECOG performance status			
ECOG 0–1	195 (85%)	128 (91%)	0.06
ECOG 2–3	31 (13%)	13 (9%)	
Child–Pugh Class			
A	183 (82%)	122 (87%)	0.46
B	39 (18%)	18 (13%)	
BCLC stage			
B	21 (9%)	19 (13%)	0.20
C	209 (91%)	123 (87%)	
ALBI grade			
Grade 1	48 (21%)	49 (35%)	**0.02**
Grade 2	169 (75%)	89 (63%)	
Grade 3	9 (4%)	3 (2%)	

*Note*: Bold values indicate statistical significance of *p*<0.05.

Abbreviations: ALBI, albumin‐to‐bilirubin; ECOG, Eastern Cooperative Oncology Group; NASH, non‐alcoholic steatohepatitis; SBRT, sterotactic body radiotherapy; TACE, transarterial chemoembolization; TARE, transarterial radioembolization.

Table 2 shows the first‐line systemic treatment agents that were utilized by HCC patients in each era. There was a significant decreased proportion of patients receiving SOR from 97.4% in SOR era to 23.2% in post‐SOR era. In contrast, LEN and A + B as first‐line agents increased to 50.7% and 22.5%, respectively.

**TABLE 2:** First‐line treatment in the overall population, SOR era, and post‐SOR era.

	SOR era *n* = 230	Post‐SOR era *n* = 142
SOR	224 (97.4%)	33 (23.2%)
LEN	0 (0.0%)	72 (50.7%)
A + B	0 (0.0%)	32 (22.5%)
Durvalumab + Tremelimumab	0 (0.0%)	2 (1.4%)
Nivolumab	1 (0.4%)	0 (0.0%)
Durvalumab	0 (0.0%)	1 (0.7%)
Other	5 (2.2%)	2 (1.4%)

### 3.2 Treatment outcomes

**FIGURE 1:**
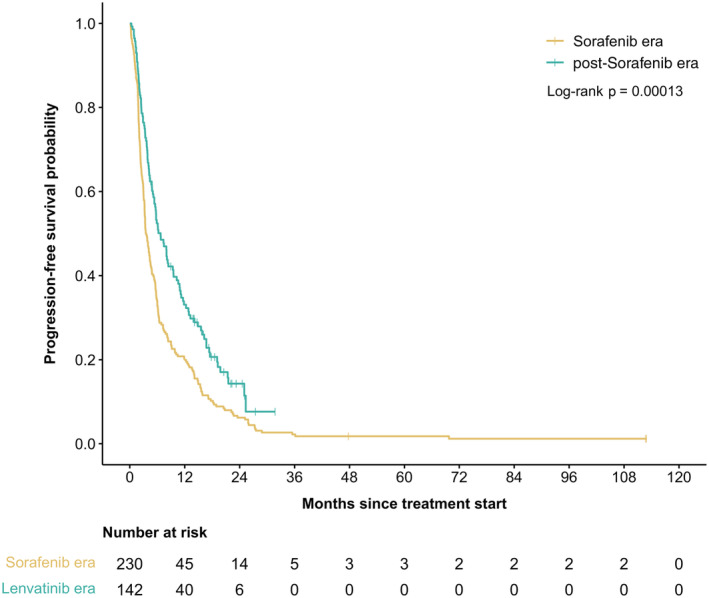
Kaplan–Meier curves for PFS according to the time era at treatment initiation.

**FIGURE 2:**
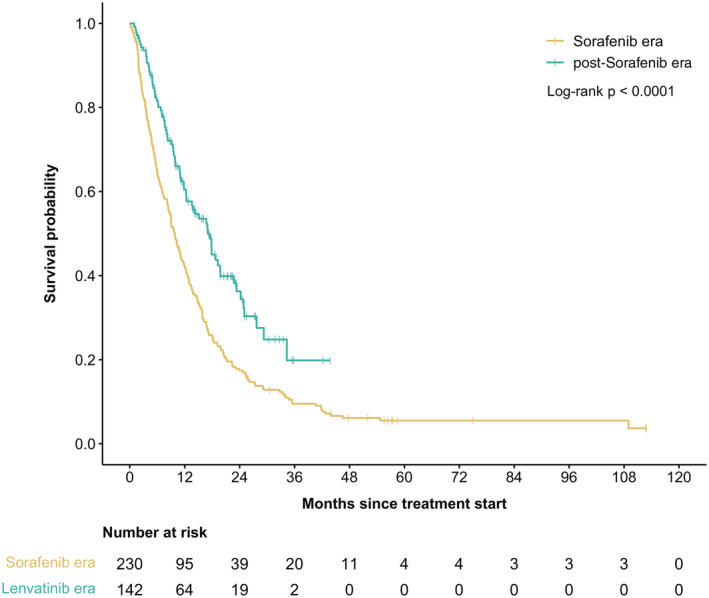
Kaplan–Meier curves for OS according to the time era at treatment initiation.

Table 3 demonstrates the outcomes and reason for discontinuation between overall population, SOR era, and post‐SOR era. Median treatment duration was 3.2 months and 4.8 months, while median follow‐up time was 9.6 and 11.0 months for SOR era and post‐SOR era, respectively. There was a statistically significant improvement in RR from 15% to 26% for SOR era and post‐SOR era (*p* = 0.02). Median PFS in SOR era was 3.8 months (95% CI: 3.2–4.6) versus 7.9 months (95% CI: 5.8–10.9) in post‐SOR era also showing significant improvement of 3.9 months (HR 0.57, 95% CI: 0.44–0.75, *p* < 0.0001; Figure 1). There was also a 7.2‐month improvement of median OS in the post‐SOR era (9.8 months vs. 17.0 months; HR 0.64, 95% CI 0.51–0.81, *p* < 0.0001; Figure 2).

**TABLE 3:** Outcomes and reason for discontinuation in the overall population, SOR era, and post‐SOR era.

	SOR era *n* = 230	Post‐SOR era *n* = 142	*p*‐value
Median treatment duration, months	3.2 (0.1–126.3)	4.8 (0.2–25.5)	**0.02**
Response rate (RR)	31 (15%)	34 (26%)	**0.02**
Median PFS (95% CI), months	3.8 (3.2–4.6)	7.9 (5.8–10.9)	**<0.0001**
Median OS (95% CI), months	9.8 (8.4–11.8)	17.0 (12.4–22.7)	**<0.0001**
Median follow‐up time	9.6	11	0.17
Reason for discontinuation			
Progression	141 (62%)	62 (50%)	**0.01**
Toxicity	52 (23%)	48 (39%)	
Patient choice	14 (6%)	8 (7%)	
Death	19 (8%)	5 (4%)	

*Note*: Bold values indicate statistical significance of *p*<0.05.

Worsening Child–Pugh class scores from the initiation to the completion of first‐line treatment were more frequently observed in the SOR era cohort compared to the post‐SOR era cohort; transitions from A to B at 32.2% versus 24.3%, A to C at 4.8% versus 3.6%, and B to C at 4.8% versus 1.8%. These comparisons were not statistically significant.

**FIGURE 3:**
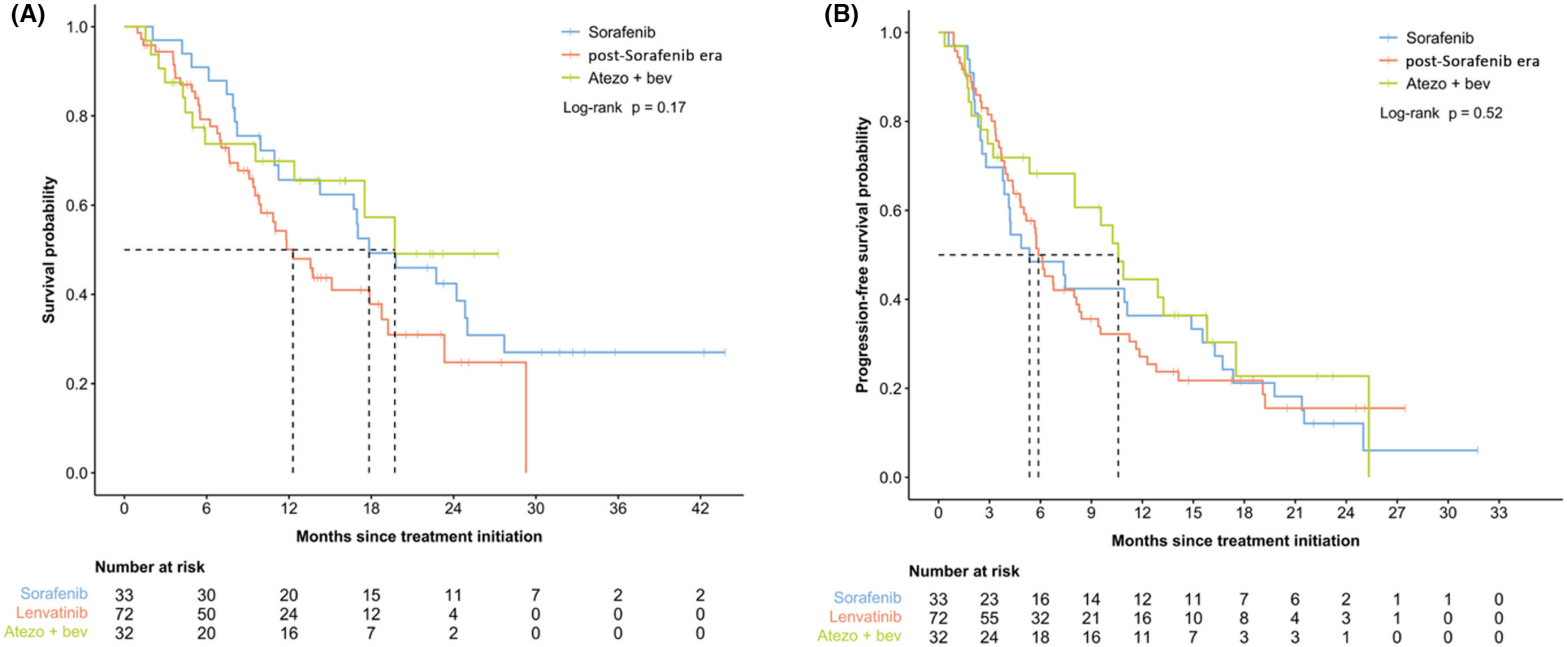
Kaplan–Meier curves for outcomes by first‐line systemic therapy regimen, among patients who initiated treatment in the post‐sorafenib era; (A) overall survival; (B) progression free survival.

Subgroup analyses were performed in the post‐SOR era cohort to examine outcomes by first‐line systemic treatments. Median OS was 19.7 months (95% CI: 12.4‐infinity) in A + B subgroup compared to 12.3 months (95% CI: 9.8–19.2) with LEN and 17.8 months (95% CI: 14.3–27.7; Figure 3A) with SOR. Median PFS was 10.6 months with A + B (95% CI: 8‐infinity) compared to 5.9 months (95% CI: 5.0–8.4) with LEN and 5.4 months (95% CI: 3.9–16.3; Figure 3B) with SOR. Table 4 contains the investigator‐assessed RR by first‐line treatment options in the post‐SOR era. We saw a trend of improvement in RR in both LEN (33%) and A + B subgroups (30%) compared to the SOR subgroup (13%) (*p* = 0.11).

**TABLE 4:** Response rate by 1 L treatment options in the post‐SOR era.

	Overall *n* = 137	Sorafenib *n* = 33	Lenvatinib *n* = 72	Atezo‐Bev *n* = 32	*p*‐value (SOR vs. LEN + A‐B)
Response rate (RR)	34 (27%)	4 (13%)	21 (33%)	9 (30%)	0.11
Best radiographic response					
Complete response	1 (1%)	0 (0%)	1 (2%)	0 (0%)	0.21
Partial response	33 (26%)	4 (13%)	48 (39%)	9 (30%)	
Stable disease	47 (38%)	13 (42%)	8 (7%)	14 (47%)	
Progressive disease	44 (35%)	14 (45%)	5 (4%)	7 (23%)	

## 4 DISCUSSION

This study was able to show superior efficacy outcomes of advanced HCC patients treated in the post‐SOR era, where patients were predominantly given (A + B) or LEN, compared to the SOR era. To the best our knowledge, this is the first study to examine the efficacy of various systemic treatments between two time periods within advanced HCC population in the real world. The median OS of patients in the SOR era was 9.8 months which is slightly worse than the OS reported in the SHARP trial (median OS of 10.7 months).^3^ This may be secondary to higher percentage of Child–Pugh class B and BCLC C stage compared to SHARP trial. We also included patients with poor ECOG PS, reflecting real‐world population that would have been excluded in the SHARP trial. The median OS of patients treated in the post‐SOR era are much higher at 17.0 months. This is expected as the median OS from the A + B arm of the IMBrave150 trial was 19.2 months^4^ and the LEN arm of the REFLECT trial was 13.6 months. As expected, PFS was also significantly higher for the patients treated in the post‐SOR era compared to SOR era (7.9 months vs. 3.8 months, respectively). The post‐SOR first‐line treatment PFS of 7.9 months was similar to the REFLECT trial LEN arm PFS of 7.4 months and IMbrave150 A + B arm PFS of 6.9 months.^4^, ^5^ This is not surprising since most patients in the post‐SOR era received LEN and several received A + B as first‐line therapy. The PFS of 3.8 months that we observed in the SOR era cohort appears reasonable, but PFS was not reported in the SHARP trial. However, in that study time to radiographic progression in the SOR arm was noted to be 5.5 months.

In this study, RR increased from 15% in the SOR era to 26% in the post‐SOR era, however, this RR was based on the physicians' assessment of CT and/or MRI results and not RECIST 1.1 or mRECIST criteria. It is noteworthy that, despite the observed worsening of Child–Pugh class scores, the RR documented in this study is comparable to the objective response rates reported in both the LEN arm of the REFLECT and A + B arm of the IMbrave150 trials.^4^, ^5^

With respect to the post‐SOR era cohort, we performed a subgroup analysis based on first‐line treatment regimen which identified A + B as a potential driver for improved outcomes. Although statistically not significant, A + B (*n* = 32) had higher PFS and OS than LEN (*n* = 72) and SOR (*n* = 33). We also noted a trend of improved RR in A + B and LEN cohort compared to SOR in this subgroup analyses. Recent literature in a retrospective cohort showed similar trends. Hiraoka et al. retrospectively compared A + B to LEN in real‐world Japanese cohort and found statistically improved PFS and OS, but no difference between ORR per mRECIST.^10^ In a non‐viral combined European and East Asian cohort, however, there was a significant higher OS and PFS in LEN compared to A + B.^11^ Interestingly, our subgroup analyses identified SOR cohort within post‐SOR era exhibiting mOS of 17.8 months. The improved OS compared to REFLECT trial's SOR arm may be secondary to earlier switch to second‐line agents such as LEN due to poor tolerability,^5^ hence with a possible additional OS benefit from a second‐line TKI. The recent phase III COSMIC‐312 trial which compared advanced HCC patients receiving cabozatinib + atezolizumab to SOR as first‐line systemic treatment also showed a longer than expected median OS of 15.4 months in the SOR arm, and possibly these patients benefited from subsequent systemic treatments.^8^

Our study appears to show increased clinical benefit with the introduction of A + B and LEN as first‐line treatment options for advanced HCC compared to the SOR era; however, these treatments do come at an increased cost. In a cost‐effective analysis of first‐line treatments for advanced HCC in China and the United States, LEN was found to be the most cost‐effective strategy.^14^ In a separate cost‐effectiveness study by Zhang et al. A + B, particularly, while being clinically beneficial, was found not be cost‐effective from a US payer perspective.^15^

We also noted that there was a higher rate of discontinuation of first‐line treatment due to toxicity in the post‐SOR era. The reason for this is not entirely clear as LEN and A + B are usually noted to be better tolerated than SOR as noted in the IMBrave150 and REFLECT trials.^3^, ^4^ Perhaps patients were less willing to put up with toxicities of treatment since in the post‐SOR era there are more systemic treatment options available after first‐line. Further, clinicians may be more inclined to change therapies in case of toxicities due to the multiple available treatment options.

Our study also analyzed changes in Child–Pugh score from start to end of the first‐line treatment. The SOR cohort showed higher proportion of patients with worsening Child–Pugh class from diagnosis to treatment end. Due to different PFS and potential different start time since diagnosis, it is difficult to conclude that SOR cohort truly had worsening liver function from treatment. However, we infer that post‐SOR cohort had more stable liver function with various treatment that may have allowed patients to transition to subsequent lines of therapy and possibly contributed to longer median OS.

Limitations to this study include the retrospective design where the patient population can be affected by selection bias. Our study also reflects a limited follow‐up period due to the approval of LEN in 2018. Longer follow‐up periods can help clarify if there are prolonged survival from newer subsequent lines of therapy in post‐SOR era. This study also had insufficient data regarding treatment‐related adverse events as these were frequently not documented in detail in the reviewed charts, and as a result toxicity was not included as a focus of this study. However, we saw that toxicity as a reason for discontinuation was increased in the post‐SOR era. This can be explored in a future study.

In conclusion, this study affirms the substantial advancements in the real‐world treatment of advanced HCC patients with systemic therapy. The efficacy outcomes of real‐world Canadian advanced HCC patients appear to closely reflect those seen in clinical trials. LEN and the recently introduced A + B do appear to be a main contributor to these improved outcomes.

## AUTHOR CONTRIBUTIONS

**Chloe A. Lim:** Data curation (equal); investigation (equal); methodology (equal); project administration (lead); writing – original draft (lead); writing – review and editing (equal). **Carla P. Amaro:** Conceptualization (equal); data curation (equal); methodology (equal); resources (equal); writing – review and editing (equal). **Philip Q. Ding:** Formal analysis (lead); software (equal); validation (equal); writing – review and editing (equal). **Winson Y. Cheung:** Conceptualization (equal); funding acquisition (equal); supervision (equal). **Vincent C. Tam:** Conceptualization (lead); data curation (equal); funding acquisition (equal); supervision (lead); validation (equal); visualization (equal); writing – review and editing (equal).

## CONFLICT OF INTEREST STATEMENT

Vincent C. Tam has served as a consultant or advisor for AstraZeneca and Incyte, has received honoraria from AstraZeneca, BMS, Eisai, Incyte, Ipsen, Merck and Roche, and has received research funding from AstraZeneca, Eisai, Ipsen and Roche.

## ETHICS STATEMENT

The study was conducted in accordance with the Declaration of Helsinki, and approved by the Health Research Ethics Board of Alberta (HREBA.CC‐15‐0042). A waiver/exempt was granted by the IRB/Ethics Committee for written consent by human participants.

## Data Availability

The data presented in this study are available on request from the corresponding author.
